# Using Simulation Training to Promote Nurses’ Effective Handling of Workplace Violence: A Quasi-Experimental Study

**DOI:** 10.3390/ijerph16193648

**Published:** 2019-09-28

**Authors:** Jin-Lain Ming, Hui-Mei Huang, Shiao-Pei Hung, Ching-I Chang, Yueh-Shuang Hsu, Yuann-Meei Tzeng, Hsin-Yi Huang, Teh-Fu Hsu

**Affiliations:** 1 Department of Nursing, Taipei Veterans General Hospital, No.201, Sec. 2, Shipai Rd., Beitou District, Taipei City 11217, Taiwan; hmhuang2@vghtpe.gov.tw (H.-M.H.); sphong@vghtpe.gov.tw (S.-P.H.); Cichang2@vghtpe.gov.tw (C.-I.C.); ys_hsu@vghtpe.gov.tw (Y.-S.H.); ymtzeng@vghtpe.gov.tw (Y.-M.T.); 2 Department of Biostatistics Task Force, Taipei Veterans General Hospital, No.201, Sec. 2, Shipai Rd., Beitou District, Taipei City 11217, Taiwan; sweethsin509@gmail.com; 3 Emergency Department, Taipei Veterans General Hospital, No.201, Sec. 2, Shipai Rd., Beitou District, Taipei City 11217, Taiwan; tfhsu@vghtpe.gov.tw

**Keywords:** workplace violence, simulation training, nurse

## Abstract

*Background*: Workplace violence in the health care sector has become a growing global problem. Research has shown that although caregivers comprise a high-risk group exposed to workplace violence, most of them lacked the skills and countermeasures against workplace violence. Therefore, through a quasi-experimental design, this study aimed to investigate the effectiveness of situational simulation training on the nursing staffs’ concept and self-confidence in coping with workplace violence. *Methods*: Workplace violence simulation trainings were applied based on the systematic literature review and the conclusions from focus group interviews with nursing staff. Data were obtained from structured questionnaires including: (1) baseline characteristics; (2) perception of aggression scale (POAS); and (3) confidence in coping with patient aggression. *Results*: The results revealed that training course intervention significantly improved the nursing staffs’ self-perception and confidence against workplace violence (*p* < 0.001). *Conclusions*: The “simulation education on workplace violence training” as the intervention significantly improved the workplace violence perception and confidence among nursing staffs in coping with aggression events.

## 1. Background

### 1.1. Workplace Violence among Health Care Workers

Physical violence, injury, harassment, and bullying at workplaces are not new phenomena worldwide [1]. Nelson, a leading expert on workplace violence, has pointed out that in both developed and undeveloped countries, the rate of violence and abuse towards health workers was four times higher than that towards other occupational groups [2]. In particular, health workers directly facing the patients and public are common victims of workplace violence. Prior research has shown that female nurses were the most frequent group at high risk of workplace violence [3,4,5]. An increasing incidence of workplace violence in the health environment has raised a great concern among workers and employers [3,4,6,7]; the incidence of violent episodes has been recognized as a major health priority by the World Health Organization (WHO) and the International Council of Nurses (ICN) [8] and a “zero tolerance” policy for workplace violence was then proposed [9]. Based on the experiences from prior studies, identifying the factors which lead to workplace violence, could help facilitate documenting and reporting violent episodes as well as developing effective prevention strategies to protect nursing staff from exposure to workplace violence [10,11].

### 1.2. Workplace Violence Training Program and Simulation Teaching

In terms of developing well-designed workplace violence training programs for health care workers, the most significant gap in content was the lack of attention to risk assessment and policy for workplace violence. Simulation is defined as learning activities in a range of mimicked real-world clinical environments, through role play or immersive devices, to reach the goal of demonstration procedures, decision-making, and critical thinking [12,13,14]. Simulation education is more and more frequently used in nursing education and is considered as an effective clinical teaching strategy [14]. As the importance of simulation training has grown, many studies have demonstrated the effectiveness of simulation teaching strategies [12]. However, studies investigating the effects of simulation training on workplace violence prevention are still limited. Although prior studies have reported the prevalence of workplace violence [2,6,7] and the effectiveness of prevention training courses in improving workplace violence awareness and reducing violence incidence [15,16,17,18,19,20,21,22], there have been few studies performing comprehensive analysis in studying the effectiveness of violence education and training strategy among nursing staff in Taiwan. This study aimed to investigate the perception of workplace violence and the effectiveness of self-confidence in coping with aggression events through simulation training and education for generalist nurses.

## 2. Materials and Methods

### 2.1. Setting and Participants

In a quasi-experimental study, a total of 66 participants were enrolled via convenience sampling in a nearly 3000-bed national medical center in Taipei, Taiwan. Participants were nursing staff members working in the units with high risk of workplace violence exposure, including emergency departments and medical and surgical wards. Participants were invited to participate in 3-h courses for “simulation education on workplace violence training”, and to complete the paper questionnaires both before and immediately after all training courses were completed. The participants were interviewed by using three structured questionnaires, including: (1) basic information; (2) perception of aggression scale (POAS); and (3) confidence in coping with patient aggression.

This study was approved by the Institutional Review Board Taipei Veterans General Hospital (VGHIRB No. 2015-06-007A). The aim of this study, procedures, methodology, and the subject’s rights were well explained to the eligible nursing staff according to the informed consent form. Written informed consent was obtained from each participant before data collection.

### 2.2. Training Program and Simulation Teaching

The content design of the “simulation education on workplace violence training” was based on the systematic reviews and nursing staff focus group interviews for framework development of workplace violence training [10,14,23,24,25]. The courses were designed to simulate verbal and physical violence in the real-workplace scenarios, prompting the participants to construct a problem-solving strategy during the learning process and to apply the strategy to the relevant situations.

The details of the courses are shown in Table 1. The teaching elements include the introduction of workplace violence, simulation-based communication, real case and group discussions, videotaped simulations of real case scenario, coping strategy, and discussions after watching the video. The participants were divided into four groups (eight to nine persons in each group) for simulation exercises. A total of two simulation training courses were scheduled. Two expert focus group interviews were arranged to examine the content validity of the courses. 

The film was organized based on the real situation, and was shot, edited, and compiled into teaching video according to the principles for simulation design proposed by Jeffries (2007) [26]: (1) introduction; (2) learning objective; (3) case study; and (4) assessment of countermeasures.

### 2.3. Study Instruments

#### 2.3.1. Baseline Characteristics

Baseline characteristics of age, gender, education level, marital status, professional title, nursing level, work experience, current unit, current department, seniority, workplace violence experience, etc., were collected for identifying the risk factors for workplace violence (Supplementary Materials).

#### 2.3.2. Perception of Aggression Scale (POAS)

The POAS was constructed by Jansen et al. to assess nurses’ attitudes toward workplace aggression [27]. The original 32-item POAS was reduced to 12 items by Needham et al. (2004), and consists of two dimensions [28]. The first dimension (Dimension 1) regards violence as a dysfunctional/undesirable phenomenon, and the second dimension (Dimension 2) regards violence as a functional/comprehensible phenomenon, representing negative (Dimension 1) and positive (Dimension 2) perceptions of aggression, respectively. The questionnaire was answered with the five-point Likert scale with good reliability and validity (reliability coefficient α of r = 0.69 and 0.67, retest reliability was r = 0.76 and 0.77 for factors 1 and 2, respectively). The higher scores indicate that the participants had clearer concepts of workplace violence, and the scale is widely used in workplace violence research [28].

#### 2.3.3. Confidence in Coping with Patient Aggression 

The scale developed by Thackrey is used to measure confidence in dealing with patient aggression [29]. The scale consists of 10 items to be answered using the five-point Likert scale. The higher scores indicate higher confidence in coping with patient aggression. The scale had good α reliability (overall Cronbach’s α: 0.92), and it is widely used in workplace violence studies.

### 2.4. Statistical Analyses

Descriptive analyses were performed for category variables such as gender, education level, marital status, job title and level, work experience, current unit, current department, and workplace violence experience; these variables were described by frequency distribution and percentage. Continuous variables such as age and seniority were described by mean and standard deviation (STD). Differences between pre- and post-test performance of workplace violence perception and self-confidence courses were compared using generalized estimating equations (GEE). Data analyses were performed by using the software SPSS for Windows V23.0.

## 3. Results

### 3.1. Baseline Characteristics

The average age of the 66 participants was 32 ± 9 years old, most of them were female (*n* = 58, 87.9%), single (*n* = 44, 66.7%), with a bachelor’s degree (*n* = 57, 86.4%). The majority of participants in this study were registered nurses (RNs, *n* = 27, 40.9%) and level-2 nurses (N2s, *n* = 29, 43.9%). As for unit types, 29 participants worked in the emergency department (43.9%) and 37 participants worked in medical and surgical wards (56.1%). A total of 44 (66.7%) participants reported that they had experienced workplace violence during nursing work. A total of 40 (60.6%) participants had never received any workplace violence training courses before enrollment, and 58 (87.9%) reported that they would like to receive relevant workplace violence courses and training. The total work experience was 10.1 ± 8.9 years on average, and the average current work experience in the medical center was 5.3 ± 5.2 years (Table 2).

### 3.2. Evaluation of the Outcome of Workplace Violence Simulation Training Course Intervention

Regarding the pre-test scores, the average score of workplace violence perception (negative) before simulation training intervention was 25 ± 3, the attitude (positive) score was 21 ± 4, and the confidence in coping skills score was 28 ± 4 (Table 3). After simulation training intervention, the post-test score of workplace violence perception (negative) was 26 ± 3, the attitude (positive) score was 23 ± 4, and the confidence in coping skills score was 32 ± 5 (Table 3).

In the GEE analyses for comparing the differences between the pre- and post-test performances of workplace violence perceptions, a single variant analysis was firstly carried out for independent variables such as age, gender, marital status, education level, job title, level, work unit, work experience, total work experience, seniority, experience of workplace violence, experience of workplace violence training, and intention to receive workplace violence training. Significant differences were found in marital status, education level, nursing level, experience of workplace violence, and experience of workplace violence training (*p* < 0.05). After multivariable adjustment, post-test performances of factor 1 and factor 2 reached statistical significance (*p* < 0.001), and the score increased by 1.05 and 1.44 points in factors 1 and 2, respectively, as compared to pre-test performances (Table 4). Nursing levels of N2 and N3 were scored as 2.18 (*p* = 0.02) and 5.09 (*p* < 0.001) points higher than N, respectively. In addition, the scores of the participants with the experience of violence decreased by 2.45 points compared to the participants without the experience of violence (*p* < 0.001). The scores of the participants with the experience of workplace violence training decreased by 1.58 points compared to the participants without training (*p* = 0.02).

In the GEE analyses for confidence in coping skills, significant differences were found in the education level and intention to receive workplace violence training (*p* < 0.05). The GEE analyses revealed a significant increase of 3.94 points in the post-test as compared to the pre-test (*p* < 0.001; Table 5). Significant differences were also found in the education level and intention to receive workplace violence training (*p* < 0.05). The scores of the participants who graduated from graduate school (or above) and university level decreased by 5 and 3.92 points, respectively, compared to the participants with college level education (all *p* < 0.001). The scores of the participants with intention to receive workplace violence training decreased by 2.42 points compared to the participants without the intention to receive training (Table 5).

## 4. Discussion

This study has several findings, as follow: (1) The “simulation education on workplace violence training” as the intervention significantly improved the workplace violence perception and confidence in coping with aggression events. (2) Higher nursing level was associated with higher scores of workplace violence perception. In contrast, the scores of the participants with prior episodes of workplace violence and workplace violence training experiences were negatively associated with workplace violence perceptions. (3) Significant declines in confidence in coping skills were found in participants with higher education levels and the intention to receive workplace violence training.

Prior studies have provided evidence for prevention training courses improving workplace violence awareness and reducing violence incidence [15,16,17,18,19,20,21,22]. In a systematic review analyzing nine studies on the impact of workplace violence prevention training among nursing staff, all studies reported increased confidence and improved attitude, skills, and knowledge after receiving training [19]. One study discussing workplace violence prevention demonstrated the effectiveness of education and training [23]. Needham et al. (2005) designed a series of violence prevention training courses and suggested that nursing students receiving violence prevention courses had significantly higher self-efficacy and confidence in aggression management as compared to the control group [15]. Lamont and Brunero (2018) demonstrated that overall confidence in coping with workplace violence significantly increased after 78 participants completed the training courses in a tertiary referral hospital in Australia [21]. Moreover, Cook et al. performed a meta-analysis for evaluating the outcomes of simulation training, and reported that simulation training was an effective teaching strategy in terms of knowledge and satisfaction outcomes [30]. 

Simulation is a participatory experience, helping participants to build their own problem-solving strategies during the learning process, to transform and apply these strategies to relevant situations, and finally to effectively improve their levels of awareness and problem-solving capabilities [12,13,14]. An integrative review confirmed the efficacy of simulation teaching in nursing education by analyzing 160 articles [14]. A total of 68.1% studies used simulation teaching and 31.9% used training skills to develop clinical reasoning. The study reported an improved self-efficacy and self-confidence after simulation teaching. The findings of our study are compatible to the above-mentioned studies, indicating that the “workplace violence simulation training course” is applicable to improve the workplace violence-related concepts, which can enhance confidence in coping skills, and can increase confidence in dealing with aggression. 

The guideline of the Occupational Safety and Health Administration (OSHA) listed three groups of risk factors that lead to violence in health care, including: (1) clinical factors (e.g., individual pain, altered mental status, history of violence, and the influence of drugs and alcohol); (2) environmental factors (e.g., layout and design of the workspace); and (3) organizational factors (e.g., discouragement to report and difficulty in reporting violent incidents, and lack of staff training and preparedness) [10]. In addition, Magnavita et al. proposed a viewpoint for developing a violence prevention program, and emphasized that the violence prevention can not only be based on clinical or individual measurements, but all educational, organizational, and medical measures are necessary elements in developing the prevention program and reducing violent events [31].

In this study, we found that a total of 66.7% participants had experienced violent episodes during work, indicating that violent episodes of worker-to-worker or patient-to-worker violence could be very frequent. Magnavita et al. had previously reported an Italian experience that nurses were mostly assaulted or harassed by patients or their relatives, whereas younger nursing students were more frequently exposed to lateral and vertical violence from other healthcare workers [4]. In the current study, we exhibited that the concept scores of workplace violence were higher in the nursing staffs of N3 and N2 than the junior staffs of N. The reason could be that these groups had more experience in the clinical setting. Therefore, they had more positive attitudes in facing workplace aggression.

For those who had lower scores on workplace violence perceptions, with prior experiences of workplace violence and workplace violence training, we suspected that they may be affected by their previous negative thinking on workplace violence. From the findings of our study, the factors of nursing level, previous exposure to workplace violence, bullying, and previous experience of workplace violence training may affect the training performance. As a result, we should take these factors into detailed and various considerations while propagating or planning workplace violence prevention programs.

- In terms of self-confidence in coping with aggression, our study showed that higher education level was associated with lower confidence. Significantly lower confidence in coping skills was found in nursing staff with graduate or university degrees when compared to those with college degrees. Based on the fact that most of the nurses had their Bachelor’s degree, the managers should strengthen their confidence in coping with workplace violence.

### 4.1. Limitations, Strengths, and Further Research

This study is a single-group designed approach focused on the effectiveness of the intervention of “simulation education on workplace violence training” in coping with workplace violence for primary nursing staff in a medical center in Taipei. Meanwhile, since only single pre- and post-tests were conducted in this study, it is not feasible to measure the sustainability of learning effects. In addition, the outcome assessment did not include the follow-up of the occurrences of workplace violence and the actual response skills. In the present study of the intervention of “simulation education on workplace violence training”, only basic attributes were analyzed. However, there are still other variables to be discussed, such as whether there were regular environmental hazard assessments in the medical settings, or the assessment of the supervisor’s attitude toward the workplace violence incidents. On the other hand, there are many factors affecting the effectiveness of learning, such as different education and training methods, and personal prerequisite knowledge.

This was the first time that our hospital conducted a workplace violence prevention program by applying simulation training. Through 3-h training courses, the nursing staff were provided with required concepts of violence prevention, and significantly improved their concepts of workplace violence prevention and self-confidence in coping strategies. The improvement could be related to the adoption of appropriate teaching strategies: By using the systematic development of classroom courses, the use of audio-visual simulation and practical exercises could strengthen the skills of workplace violence prevention. Applying a teaching video for real-case presentation can not only increase the participants’ learning interests, but also improve the teaching quality. Besides, both the satisfaction of the course and the extent of understanding were highly evaluated, suggesting that simulation training was a good training strategy for workplace violence prevention.

In the future, we recommended collecting other subjective and objective indicators to be incorporated for the evaluation of workplace violence simulation training program, e.g., the incidences of workplace violence, the attitude of nursing staff to deal with workplace violence, and actual response skills. A clear survey on worker-to-worker/patient-to-worker or lateral/vertical violence should be conducted in the future. In addition, extending the follow-up period to understand the long-term effect of workplace violence simulation training courses could be considered. All the above-mentioned study directions may provide more comprehensive manners to build a high-quality work environment.

### 4.2. Clinical Implications

The results of this study might support the workplace violence simulation training course in the clinical environment. It is also pointed out in the literature that workplace violence prevention programs reduce the exposure of workplace violence behaviors, which in turn reduce the psychological distress of employees, and has a positive impact on the overall quality of life and working style of employees [32]. It is recommended to develop different simulations for specific medical units in the future, such as emergency, psychiatric, maintenance institutions, home care, internal medicine wards, surgery wards and outpatient clinics. Also recommended is to integrate simulation training into the regular workplace violence prevention program of the hospital, and to update the program periodically to meet the latest needs. In the future, an online course on workplace violence prevention can be developed to facilitate more flexible learning through text, real-case videos, and interactive processes, for nursing staff to learn the prevention strategies online and practice in the real-world simulation training courses.

## 5. Conclusions

In this study, we constructed the “Workplace Violence Simulation Training Course” and investigated the training effects on the concepts of workplace violence and self-confidence in coping strategies. We concluded that significant increases in post-test scores for both perception of workplace violence and confidence against aggression events were found, indicating simulation education can improve the concepts of workplace violence and confidence in dealing with violent events.

Here, we put forward the following recommendations based on our study findings for consideration: (1) planning of a workplace violence prevention program which might incorporate simulation teaching and practice, in order to provide real-world experiences of learning and coping skills, and to increase learning interest and motivation; (2) developing and adjusting the medical unit-specific simulation training courses and integrated simulation trainings into the regular workplace violence prevention program; and (3) developing an online course based on the workplace violence prevention program to facilitate more flexible learning.

## Figures and Tables

**Table 1:** Content of the workplace violence simulation training course.

Content of the Course		Methodology	Duration
1	Review of workplace violence prevention (Recognition of workplace harassment, high-risk assessment and identification, prevention, coping, related regulations and physical and psychological adjustment)	Explanation in the classroom	60 min
2	Workplace violence simulation video (including verbal and physical violence)	Video watching	10 min
3	Prevention, handling, and communication skills for verbal abuse	Explanation and demonstration in the classroom	30 min
4	Prevention, handling, and breakaway skills for physical violence	Explanation and demonstration in the classroom	30 min
5	Group simulation exercises and group scenario review discussion	Simulation, exercise group discussion, and feedback	50 min

**Table 2:** Participants’ sociodemographic characteristics.

Item	Total *N* = 66
Age (years)	32 ± 9
Total work experience (years)	10.1 ± 8.9
Seniority (years)	5.3 ± 5.2
Gender	
Males	8 (12.1%)
Females	58 (87.9%)
Marital status	
Single	44 (66.7%)
Married	21 (31.8%)
Divorced/widowed	1 (1.5%)
Education level	
College	1 (1.5%)
University	57 (86.4%)
Graduate school	8 (12.1%)
Job title	
Registered profession nurse (RPN)	20 (30.3%)
Registered nurse (RN)	27 (40.9%)
Contract nurse	19 (28.8%)
Nursing level	
N	10 (15.2%)
N1	9 (13.6%)
N2	29 (43.9)
N3	10 (15.2%)
N4	8 (12.1%)
Work unit	
Emergency Department	29 (43.9%)
Medical and surgical wards	37 (56.1%)
Experienced workplace violence	
Yes	44 (66.7%)
No	22 (33.3%)
Received a workplace violence training course	
Yes	26 (39.4%)
No	40 (60.6%)
Wishes to take workplace violence courses and training	
Yes	58 (87.9%)
No	8 (12.1%)

**Table 3:** Scores of workplace violence perceptions and self-confidence in coping with aggression.

Items	Pre-Test	Post-Test
Attitude-factor 1 (negative)	25 ± 3	26 ± 3
Attitude-factor 2 (positive)	21 ± 4	23 ± 4
Confidence in coping skills	28 ± 4	32 ± 5

**Table 4:** Comparison of pre- and post-test of workplace violence perceptions.

Variable	Estimated Value	Standard Error	*p*-Value
Attitude-factor 1 (negative)			
Post-test vs. Pre-test	1.05	0.32	<0.001
Attitude-factor 2 (positive)			
Post-test vs. Pre-test	1.44	0.38	<0.001
Marital status			
Divorce/widowed vs. Single	−2.02	1.30	0.12
Married vs. Single	-0.10	0.81	0.90
Education level			
Graduate school (or above) vs. College	−0.79	1.45	0.59
University vs. College	−0.15	1.05	0.88
Nursing level			
N1 vs. N	1.64	1.29	0.20
N2 vs. N	2.18	0.92	0.02
N3 vs. N	5.09	1.16	<0.001
N4 vs. N	1.82	1.23	0.14
Experience of workplace violence			
Yes vs. No	−2.45	0.77	<0.001
Experience of workplace violence training			
Yes vs. No	−1.58	0.69	0.02

**Table 5:** Comparison of pre- and post-test of confidence in coping skills.

Variable	Estimated Value	Standard Error	*p*-Value
Post-test vs. Pre-test	3.94	0.66	<0.001
Education level			
Graduate school (or above) vs. College	−5.00	1.27	<0.001
University vs. College	−3.918	0.55	<0.001
Intention to receive workplace violence training			
Yes vs. No	−2.42	1.15	0.04

## Supplementary Materials

The following are available online at https://www.mdpi.com/1660-4601/16/19/3648/s1, Supplementary Material: Questionnaires.
