# Supplementary material for: Using Simulation Training to Promote Nurses’ Effective Handling of Workplace Violence: A Quasi-Experimental Study
Source: Int J Environ Res Public Health. 2019 Sep 28;16(19):3648. doi: 10.3390/ijerph16193648 (PMC6801794; doi:10.3390/ijerph16193648)
Supplement: Supplementary file 1 [file ijerph-16-03648-s001.zip › ijerph-549671-supplementary.docx]

Supplementary Materials

Questionnaire 1: Basic information.

| Participants’ sociodemographic characteristics | |
| --- | --- |
| 1 | What is your age? ____________ |
| 2 | What is your gender?   Female  Male |
| 3 | What is your marital status?   Single  Married  Divorced/widowed |
| 4 | What is your education level?   College  University  Graduate school |
| 5 | What is your current job title?   Registered profession nurse (RPN)  Registered nurse (RN)  Contract nurse |
| 6 | What is your current nursing grade?   N  N1  N2  N3  N4 |
| 7 | Which clinical unit do you currently work in?   Emergency Department  Medical wards  Surgical wards |
| 8 | How long have you worked in hospital ( in years)? ___________ |
| 9 | How long have you worked on your current unit (in years)? _______________ |
| 10 | Have you ever experienced from workplace violence?   Yes  No |
| 11 | Have you received a workplace violence training course?   Yes  No |
| 12 | Do you want to take workplace violence courses and training?   Yes  No |

|  |
| --- |

Questionnaire 2: Perception of aggression scale (POAS)

| 12 items | | **Strongly agree** | **Agree** | **Uncertain** | **Disagree** | **Strongly disagree** |
| --- | --- | --- | --- | --- | --- | --- |
| 1 | Is violent behavior toward others and self? | **1** | **2** | **3** | **4** | **5** |
| 2 | Is emotionally letting off steam? | **1** | **2** | **3** | **4** | **5** |
| 3 | Is essentially beating up someone else? | **1** | **2** | **3** | **4** | **5** |
| 4 | Offers new possibilities in nursing care. | **1** | **2** | **3** | **4** | **5** |
| 5 | Is hurting others mentally or physically? | **1** | **2** | **3** | **4** | **5** |
| 6 | Is any action of physical violence? | **1** | **2** | **3** | **4** | **5** |
| 7 | Helps the nurse see the patient from another point of view. | **1** | **2** | **3** | **4** | **5** |
| 8 | Is an expression of emotions, just like laughing or crying? | **1** | **2** | **3** | **4** | **5** |
| 9 | Is a tool that patients use to exercise power over others? | **1** | **2** | **3** | **4** | **5** |
| 10 | Is the protection of one’s own territory and privacy? | **1** | **2** | **3** | **4** | **5** |
| 11 | Is the start of more positive nurse-patient relationship? | **1** | **2** | **3** | **4** | **5** |
| 12 | Is any expression that makes someone else feel unsafe, threatened or hurt? | **1** | **2** | **3** | **4** | **5** |

Questionnaire 3: Confidence in coping with patient aggression

|  |  | **Strongly agree** | **Agree** | **Uncertain** | **Disagree** | **Strongly disagree** |
| --- | --- | --- | --- | --- | --- | --- |
| 1 | How comfortable are you working with an aggressive patient? | **1** | **2** | **3** | **4** | **5** |
| 2 | How good is your present level of training for handling psychological aggression? | **1** | **2** | **3** | **4** | **5** |
| 3 | How able are you to intervene physically with an aggressive patient? | **1** | **2** | **3** | **4** | **5** |
| 4 | How self assured do you feel in the presence of an aggressive patient? | **1** | **2** | **3** | **4** | **5** |
| 5 | How able are you to intervene psychologically with an aggressive patient? | **1** | **2** | **3** | **4** | **5** |
| 6 | How good is your present level of training for handling physical aggression? | **1** | **2** | **3** | **4** | **5** |
| 7 | How safe do you feel around an aggressive patient? | **1** | **2** | **3** | **4** | **5** |
| 8 | How effective are techniques you know for dealing with aggression? | **1** | **2** | **3** | **4** | **5** |
| 9 | How able are you to meet the needs of an aggressive patient? | **1** | **2** | **3** | **4** | **5** |
| 10 | How able are you to protect yourself from an aggressive patient? | **1** | **2** | **3** | **4** | **5** |
